# Determination of serum 25-hydroxyvitamin D status among population in southern China by a high accuracy LC-MS/MS method traced to reference measurement procedure

**DOI:** 10.1186/s12986-020-0427-7

**Published:** 2020-01-17

**Authors:** Zhiliang Cai, Qiaoxuan Zhang, Ziqiang Xia, Songbai Zheng, Lilan Zeng, Liqiao Han, Jun Yan, Peifeng Ke, Junhua Zhuang, Xinzhong Wu, Xianzhang Huang

**Affiliations:** 1 Department of Laboratory Medicine, The Second Affiliated Hospital of Guangzhou University of Chinese Medicine, Guangzhou, China; 2 Second Clinical Medical College, Guangzhou University of Chinese Medicine, Guangzhou, 510120 China; 3 Guangzhou Huayin Medical Laboratory Center, Guangzhou, China

**Keywords:** 25-hydroxyvitamin D, LC-MS/MS, PTH

## Abstract

**Objective:**

We aimed to describe the 25-hydroxyvitamin D (25(OH)D) status of southern Chinese individuals by a high-accuracy liquid chromatography tandem mass spectrometry (LC-MS/MS) method which can trace to reference measurement procedure.

**Materials and methods:**

From January 2018 to June 2019, a total of 4775 southern Chinese individuals were evaluated in our study. The serum levels of parathyroid hormone (PTH) were detected simultaneously in 162 cases. 25(OH)D was determined by LC-MS/MS, and PTH was detected using routine automated analysers. The distribution of the concentration, prevalence and seasonal variability of 25(OH)D in males and females of different age groups were studied.

**Results:**

The mean 25(OH)D concentration in our study was 32.57 ng/mL (4.20–101.40 ng/mL). The global 25(OH)D concentration in males was higher than that in females of different age group. The prevalence of vitamin D deficiency (< 20 ng/mL) in females (16.65%) was higher than that in males (6.83%). The prevalence of vitamin D deficiency (< 20 ng/mL) was most common in winter (22.98% of all women and 15.49% of all men). 25(OH)D concentrations were higher in those from whom blood samples were collected in summer and autumn than in winter and spring. 25(OH)D_2_ was detected in 672 serum samples (14.07%). In addition, there was a negative correlation between the concentrations of 25(OH)D and serum PTH (*r* = − 0.149, *P* < 0.05).

**Conclusion:**

Our study demonstrated that the average serum 25(OH)D concentration in southern Chinese individuals was higher than that in other Chinese cohorts by a high-accuracy LC-MS/MS method. The global 25(OH)D concentration in males was higher than that in females of different ages, and the prevalence of vitamin D deficiency in females was higher than that in males. Seasonal change was an important aspect of 25(OH)D concentration in young and middle-aged people but became less relevant for that in older subjects. 25(OH)D_2_ detection was of minor practical significance in our study. In addition, we also found that there was a negative correlation between the serum levels of 25(OH)D and PTH in southern Chinese individuals.

## Introduction

Vitamin D is considered to be a class of steroid hormones, and the most important members of its family are 25-hydroxyvitamin D_3_ (25(OH)D_3_) and 25-hydroxyvitamin D_2_ (25(OH)D_2_). In addition, 25(OH)D_3_ and 25(OH)D_2_ are the active forms of vitamin D [1]. 25(OH)D_3_ is mainly produced in the skin through the transformation of 7-dehydrocholesterol by UV light. 25(OH)D_2_ is primarily from plant and fungal sources [2]. Vitamin D plays a primary physiological role in maintaining the stability of serum calcium concentrations and bone health in the human body [3]. However, several studies have shown that there is a high prevalence of vitamin D deficiency in many countries around the world [4, 5]. Ming-Jse Lee et al. reported that 22.4% of the northern Taiwan population had a vitamin D deficiency [6]. Akhtar et al. concluded that vitamin D deficiency was highly prevalent among the South Asian population, including that of India, Pakistan, Bangladesh and Sri Lanka [7]. Silvia Giulianil found that approximately 30% of the population had a deficient 25(OH)D concentration in a large Southern European population [8]. Reports on the vitamin D status in Chinese populations come from Hong Kong, Taiwan and northeastern China populations [6, 9, 10], but there are few studies on the current status of vitamin D in South Chinese individuals, who have a different diet, lifestyle and climate than those of northeastern China populations.

Several factors may affect the production of vitamin D in the skin, including ageing, gender, geographical location, season, race, outdoor activities and so on [11]. Vitamin D deficiency may be a risk factor for secondary hyperparathyroidism, rickets, fragility fractures, cancer, diabetes and cardiovascular disease [5, 12, 13]. Many studies have reported that vitamin D deficiency may be more pervasive than previously thought and has become a public health problem around the world [12, 14]. Therefore, awareness of the high prevalence of vitamin D deficiency has increased, which arouses an vigorous demand for 25(OH)D detection.

The increasing demands for 25(OH)D testing have forced clinical laboratories to develop accurate methods that are suitable for routine measurements. At present, the main detection methods of 25(OH)D are performed with a variety of techniques based on different measurement principles, which are high-performance liquid chromatography-ultraviolet detection (HPLC-UV), liquid chromatography-tandem mass spectrometry (LC-MS/MS), and various immunoassay methods [15, 16]. Immunoassay methods include radioimmunoassay (RIA), enzyme-linked immunosorbent assay (ELISA), chemiluminescence immunoassay (CLIA), electrochemiluminescence immunoassay (ECLIA), automatic biochemical analysis and so on. Recently and in many studies, the most commonly used analytical methods to measure 25(OH)D metabolites have been based on immunoassays [4]. However, immunoassays are largely limited by cross-reactivity of antibodies and molecular receptor recognition of the D_2_ and D_3_ forms of the 25(OH) metabolite. In contrast, the high resolution of chromatography lends its sufficient detection specificity [17]. LC-MS/MS inherently has higher sensitivity and higher recoveries than those of immunoassays and is considered to be the gold standard for vitamin D detection [16, 18]. However, although LC-MS/MS is considered to be highly specific, the accuracy of mass spectrometry detection can be affected by the analysis of isobaric compounds [19]. The best known isobaric interference of 25(OH)D_3_ is its C-3 structural analogue 3-epi-25(OH)D_3_, which can be found in large quantities of circulating [20] but does not have all the biological activities of 25(OH)D_3_. In our study, we optimized a mass spectrometric method for the detection of 3-epi-25(OH)D_3_, 25(OH)D_3_, 3-epi-25(OH)D_2_ and 25(OH)D_2_ that meets the recommended reference measurement procedure (RMP) specifications for 25(OH)D metabolites to achieve optimal authenticity and precision [21]. In addition, compared with other reference measurement procedures, the response time of the optimized mass spectrometry method for the detection of 25(OH)D is shorter than that of other RMPs [21].

The objective of our study was to evaluate the serum 25-hydroxyvitamin D status among populations in southern China by a high-accuracy LC-MS/MS method which can trace to reference measurement procedure requirements. Moreover, we studied the distribution of serum 25(OH)D concentrations in males and females of different age groups and the seasonal variability. In addition, we explored the relationship between the concentrations of serum 25(OH)D and parathyroid hormone (PTH) in this study.

## Materials and methods

### Patients

Four thousand seven hundred and seventy-five southern Chinese individuals were evaluated in our study from January 2018 to June 2019. Fasting blood samples were taken from each subject between 8:00 a.m. and 11:00 a.m. The serum samples were federally measured at the Guangdong Provincial Hospital of Chinese Medicine and Guangzhou Huayin Medical Laboratory Center. These data were collected on the same day as the patient serum samples were collected. The study was approved by the Ethics Committee of Guangdong Provincial Hospital of Chinese Medicine, China.

### Reagents and instruments

25(OH)D_3_ and 25(OH)D_2_ reference standards were purchased from Sigma-Aldrich (St. Louis, MO, USA). Reference standards were used for the calibration of 25(OH)D_2_ and 25(OH)D_3_. 25(OH)D calibration solutions, standard reference material (SRM) 972a (25(OH)D_2_ and 25(OH)D_3_, solvent-based) and SRM 972a (four levels, serum-based) were manufactured by the National Institute of Standards Technology (NIST) (Gaithersburg, MD, USA) and purchased from Haochu Biotechnology Co. Ltd. (Beijing, China). 25(OH)D_3_–23,24,25,26,27-^13^C_5_ (99 atom % ^13^C) with a purity of 98.0% and 25(OH)D_2_–6,19,19-d_3_ (99 atom % ^13^C) with a purity of 99.3% were used as the internal standards (ISs) and were obtained from Sigma-Aldrich Co. (St. Louis, MO, USA). Vitamin D_2_, vitamin D_3_, 1α,25-dihydroxyvitamin D_3_, 3-epi-25-hydroxyvitamin D_3_, 3-epi-25-hydroxyvitamin D_2_, (24R)-24,25-dihydroxyvitamin D_3_, (24S)-24,25-dihydroxyvitamin D_3_, 1α,25-dihydroxyvitamin D_2_, and formic acid (FA) were also purchased from this company. Acetonitrile, methanol and n-hexane (LC-MS grade) were obtained from Merck (Darmstadt, Germany). Anhydrous ethanol (HPLC grade) was purchased from Fisher (USA). ZnSO_4_•7H_2_O was acquired from APIS (Beijing, China). Charcoal-stripped human serum was obtained from Bioresource Technology (FL, USA). An F5 column [kinetex F5, 2.6 μm, 100 mm × 2.1 mm (i.d.)] was obtained from Phenomenex (USA). Human serum samples were from in vitro diagnostic companies having a cooperation agreement with Huayin Health (Guangzhou Huayin Laboratory Center).

### Sample preparation

First, 200 μL standard solution or serum sample was placed in a 15 mL centrifuge tube, and 10 μL internal standard solution was added to the centrifuge tube, which was vortexed for 10 s. Then, 200 μL 0.2 mmol/L zinc sulfate solution and 300 μL methanol were added to the centrifuge tube and vortexed for 30 s. A portion of 1 mL n-hexane was added and vortexed for 1 min; the sample was centrifuged at 4 °C (4000 rpm) for 5 min, the supernatant was transferred to a 15 mL centrifuge tube, it was dried with nitrogen at room temperature, and then redissolved with 200 μL acetonitrile:water (v:v = 50:50). The in-house quality control (QC) materials were produced from a mixed human serum sample and made to three different concentration levels of 25-hydroxyvitamin D (including 25(OH)D_3_ and 25(OH)D_2_): QC-low, QC-medium, and QC-high. To determine the QC values, at least two independent measurement series were analysed three times for each operation consisting of repeated preparations of standard reference material (SRM) 972a. The concentration of the QC materials was calculated according to the standard curve of SRM 972a. The values of SRM 972a consisted of four different concentrations of 25-hydroxyvitamin D (including 25(OH)D_3_ and 25(OH)D_2_) and were certified by the National Institute of Standards & Technology using ID-LC-MS/MS RMP. The experimental protocol is described in Additional file 1.

### Biochemical parameters

25(OH)D measurement by LC–MS/MS was performed on a Waters Acquity UPLC™ with a triple quadrupole mass detector (Xevo TQ-S) system with positive electrospray ionization (ESI). The serum PTH test was performed using routine automated analysers (Cobas 8000, obtain ISO15189 certification) according to the manufacturer’s instructions.

### LC-MS/MS conditions

A Waters Acquity UPLC™ with a triple quadrupole mass detector (Xevo TQ-S) system in positive electrospray ionization (ESI) mode was used for analysis. The dwell time was 52 ms for multiple reaction monitoring (MRM) mode. The transitions and conditions for the Waters Acquity Xevo TQ-S are listed in Table 1. The selected reaction monitoring transitions were m/z 383.3 → 365.1 [25(OH)D_3_], m/z 383.3 → 257.2 [3-epi-25(OH)D_3_], m/z 388.3 → 370.1[25(OH)VD_3_-^13^C_5_)], m/z 395.3 → 119.3 [25(OH)D_2_], m/z 395.3 → 377.3[3-epi-25(OH)D_2_] and m/z 398.3 → 380.3 [25(OH)VD_2_-d_3_]. The optimized instrumental settings were capillary voltage (2.6 kV), desolvation temperature (600 °C), source temperature (150 °C), desolvation gas flow (1200 L/hr), cone gas flow (150 L/hr), nebulizer (7 bar) and collision gas flow (0.14 mL/min). The desolvation gas was provided by a nitrogen generator (PEAK), while the collision gas was argon. Chromatographic separation was achieved by using a Kinetex® F5 column at 45 °C with 0.1% FA in water (mobile phase A) and 0.1% FA in methanol (mobile phase B) at a rate of 0.45 mL/min. Initially, the mobile phase composition was 35% A and 65% B and was maintained for 1.0 min. Then, mobile phase B was increased to 75% from 1.0 min to 4.5 min. The gradient was maintained at 75% B until 7.0 min. Next, a high organic phase of 98% B flushed the column from 7.1 to 8.5 min. Finally, initial conditions were restored for a total run time of 10.0 min. The temperature of the autosampler was set at 5 °C, and the injection volume was 10 μL.

**Table 1:** Conditions of Triple Quadrupole MS

Compound	Average mass	Precursor ion (m/z)	Preoduct ion (m/z)	Cone (V)	CE(V)
25(OH)VD_3_	400.6	383.3	257.2 (Q)	25	15
			365.1 (I)	24	15
25(OH)VD_3_- ^13^C_5_	405.6	388.3	370.1	26	15
25(OH)VD_2_	412.7	395.3	119.4 (Q)	26	20
			377.3 (I)	26	10
25(OH)VD_2_-d_3_	415.7	398.3	380.3	26	15

*CE* collision energy, *Q* transition used for quantification, *I* transition used for identification

### Data analysis and statistical methods

For each set of samples in this study, a typical detection series was as follows: a reagent blank (65% methanol), a seven-point calibration series (with increasing concentrations), a blank sample (spiked with ISWS), quality control (QC) materials, serum samples, and another reagent blank (65% methanol). Demographic and anthropometric statistics were expressed as the mean ± SD when appropriate. Groups were formed on the basis of gender, age and season. Demographic data were analysed using descriptive statistical tests performed with SPSS software package, version 20.0, GraphPad Prism 5 and Microsoft Excel 2007 (Microsoft, Redmond, WA, USA). The correlations were analysed between two groups using the Spearman r test. The difference comparisons between 2 groups were performed with the unpaired t-test. A value of *P* < 0.05 was considered statistically significant.

## Results

### 25(OH)D detection via an LC-MS/MS method that traces to reference measurement procedure

**Fig. 1:**
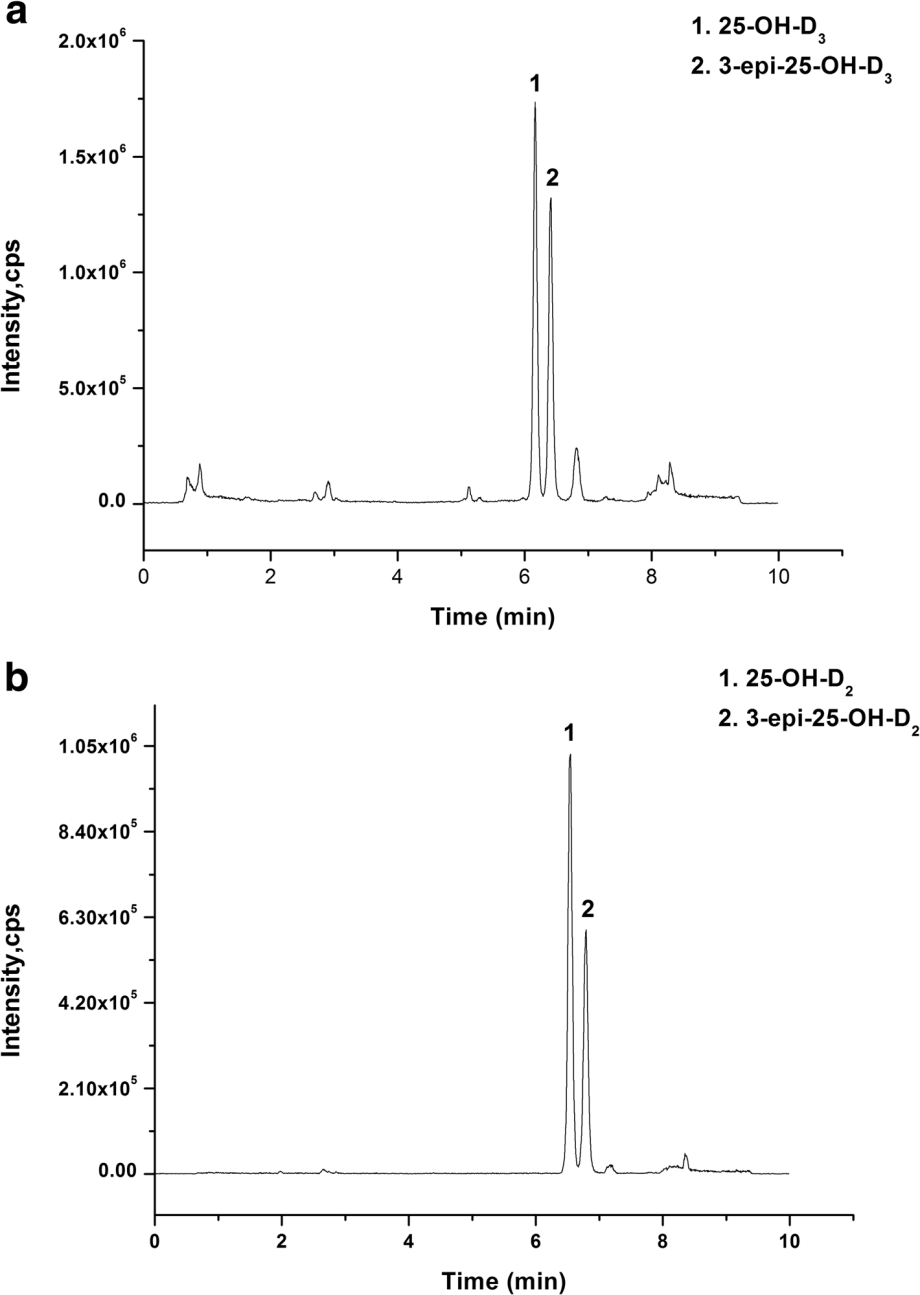
Typical selected reaction monitoring chromatogram of 10 ng/mL 25(OH)D_3_, 10 ng/mL 3-epi-25(OH)D_3_ (**a**), 10 ng/mL 25(OH)D_2_ and 10 ng/mL 3-epi-25(OH)D_2_ (**b**) in serum

The LC-MS/MS chromatographic results of SRM 972a analysed in our study at four different concentration levels of 25(OH)D_3_ and 25(OH)D_2_ are shown in Table 2 and Table 3. The biases of the four different concentration levels (Levels 1 to 4) of 25(OH)D_3_ were − 1.27, 2.94, 1.47, and 1.52%, respectively. The bias of the four different concentration levels (Levels 1 to 4) of 25(OH)D_2_ were − 4.17, 4.45, − 1.40%, and 2.26%, respectively. According to the standard curve of SRM 972a, the QC material concentration levels (Levels 1 to 3) of 25(OH)D are described in Table 4. These data indicated that the results of 25(OH)D detected by LC-MS/MS meet reference measurement procedure requirements. The optimized mass spectrometric method for the detection of vitamin D has good specificity and can selectively detect 25(OH)D_3_, 3-epi-25(OH)D_3_ (Fig. 1a), 25(OH)D_2_, and 3-epi-25(OH)D_2_ (Fig. 1b) in serum.

**Table 2:** Assessment of the bias of 25(OH)D_3_ detected by LC-MS/MS using NIST certified SRM-972a

SRM-972a 25(OH)D_3_	Certified values by NIST		LC-MS/MS		
	Target values (ng/mL)	Uncertainty	Detected values (ng/mL)	SD (*n* = 3)	Bias(%)
Level 1	28.80	1.10	28.43	0.61	−1.27
Level 2	18.10	0.40	18.63	0.34	2.94
Level 3	19.80	0.40	20.09	0.23	1.47
Level 4	28.40	0.90	29.77	0.26	1.25

*SRM* standard reference material, *NIST* National Institute of Standards & Technology

**Table 3:** Assessment of the bias of 25(OH)D_2_ detected by LC-MS/MS using NIST certified SRM-972a

SRM-972a 25(OH)D_2_	Certified values by NIST		LC-MS/MS		
	Target values (ng/mL)	Uncertainty	Detected values (ng/mL)	SD (*n* = 3)	Bias(%)
Level 1	0.50	0.06	0.48	0.07	−4.17
Level 2	0.81	0.06	0.84	0.05	4.45
Level 3	12.90	0.30	12.72	0.26	−1.40
Level 4	0.50	0.10	0.51	0.04	2.26

*SRM* standard reference material, *NIST* National Institute of Standards & Technology

**Table 4:** Assignment of quality control materials

	25(OH)D_3_		25(OH)D_2_	
	Values(${\overline{x}}_i$ ±S_i_ ng/mL)	CV(%)	Values(${\overline{x}}_i$ ±S_i_ ng/mL)	CV(%)
QC-low	28.14 ± 0.99	3.52	0.48 ± 0.02	4.82
QC-medium	34.65 ± 1.14	3.28	5.15 ± 0.22	4.31
QC-high	44.38 ± 1.29	2.91	11.44 ± 0.30	2.64

*QC* quality control, ${\overline{x}}_i$ median, *S*_*i*_ standard deviation, *CV* variable coefficient

### General characteristics and serum 25(OH)D status of the evaluated cohort

As shown in Table 5, a total of 4775 individuals from South China were included in this study. The mean age of the study population was 18.61 ± 19.88 years (1–95 years). From all 4775 serum 25(OH)D results, the proportion from males was lower than that from females: 1946 requests (40.75%) vs. 2829 requests (59.25%). Serum 25(OH)D was predominantly detected in the population between 1 and 40 years of age (87.6%). The largest age group was that of children (< 18 years) in this study (48.57%). The mean 25(OH)D concentration of the study group was 32.57 ± 11.74 ng/mL (4.2–101.4 ng/mL).

**Table 5:** 25(OH)D concentration in 4775 serum samples from South China—descriptive statistics

Sex	All		< 18 years		18-40 years		41-60 years		61-80 years		> 80 years	
	M (*n* = 1946)	F (*n* = 2829)	M (*n* = 1544)	F (*n* = 1165)	M (*n* = 119)	F (*n* = 1355)	M (*n* = 152)	F (*n* = 166)	M (*n* = 105)	F (*n* = 114)	M (*n* = 26)	F (*n* = 29)
25(OH)D(ng/mL)												
Median	34.26	29.4***	35.7	34.16**	26.07	26.43	29.66	28.29	30.5	27.2***	24.66	22.0
Mean ± SD	35.45 ± 11.71	30.60 ± 11.35	37.09 ± 11.58	35.80 ± 12.0	27.86 ± 9.29	26.91 ± 9.51	29.61 ± 10.65	28.32 ± 8.18	30.65 ± 6.62	29.49 ± 8.39	26.42 ± 9.0	24.38 ± 9.23
25th–75th	27.2–42.0	22.67–36.89	29.2–43.87	27.3–42.7	21.9–34.07	20.1–32.44	22.35–37.19	23.3–32.91	24.35–37.75	20.77–31.83	20.56–31.45	19.27–28.39

*P < 0.05, **P < 0.01, ***P < 0.001, *M* male, *F* female

### Differences in serum 25(OH)D concentration based on age and gender

**Fig. 2:**
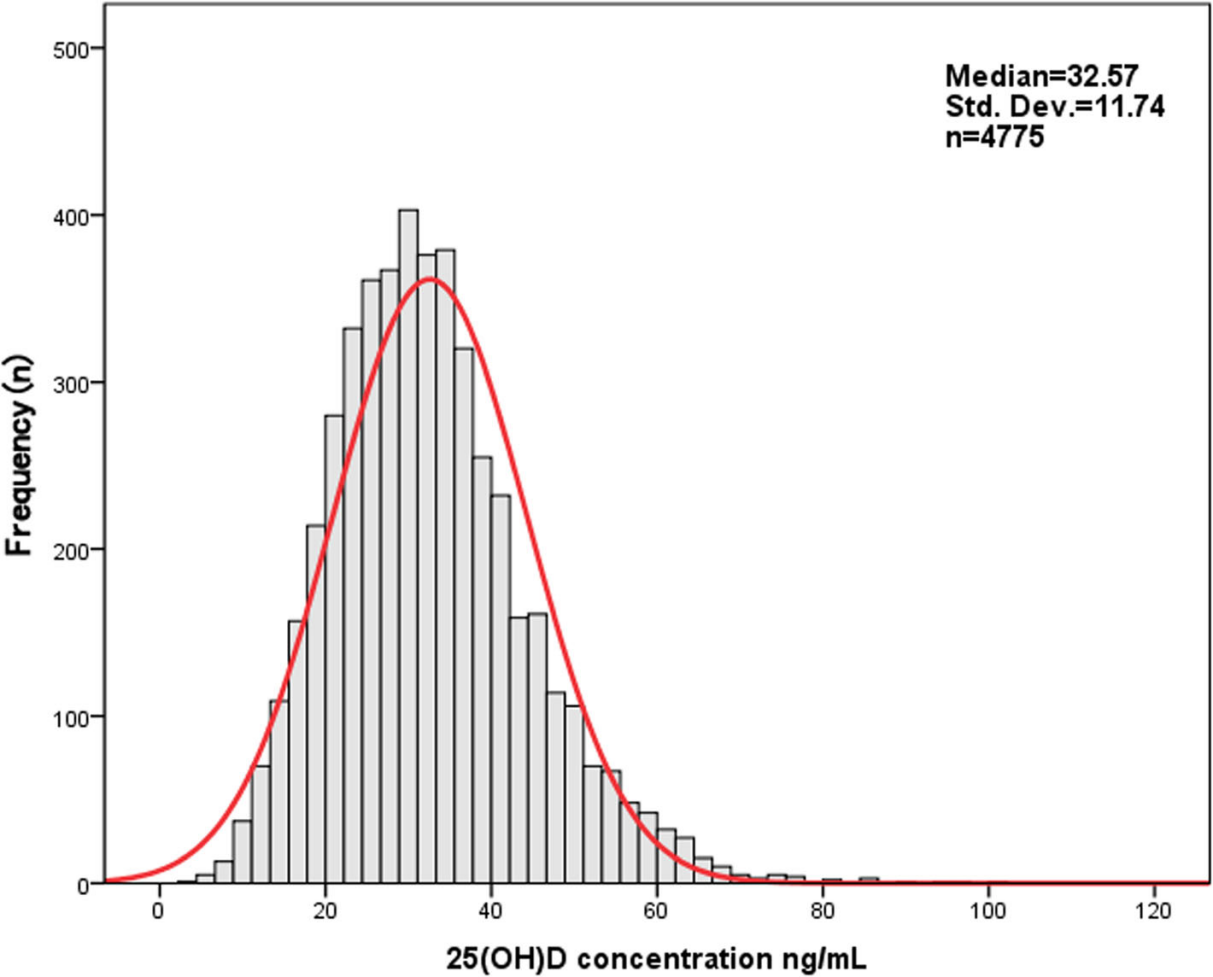
Frequency distribution of serum 25(OH)D concentrations in a southern Chinese population

**Fig. 3:**
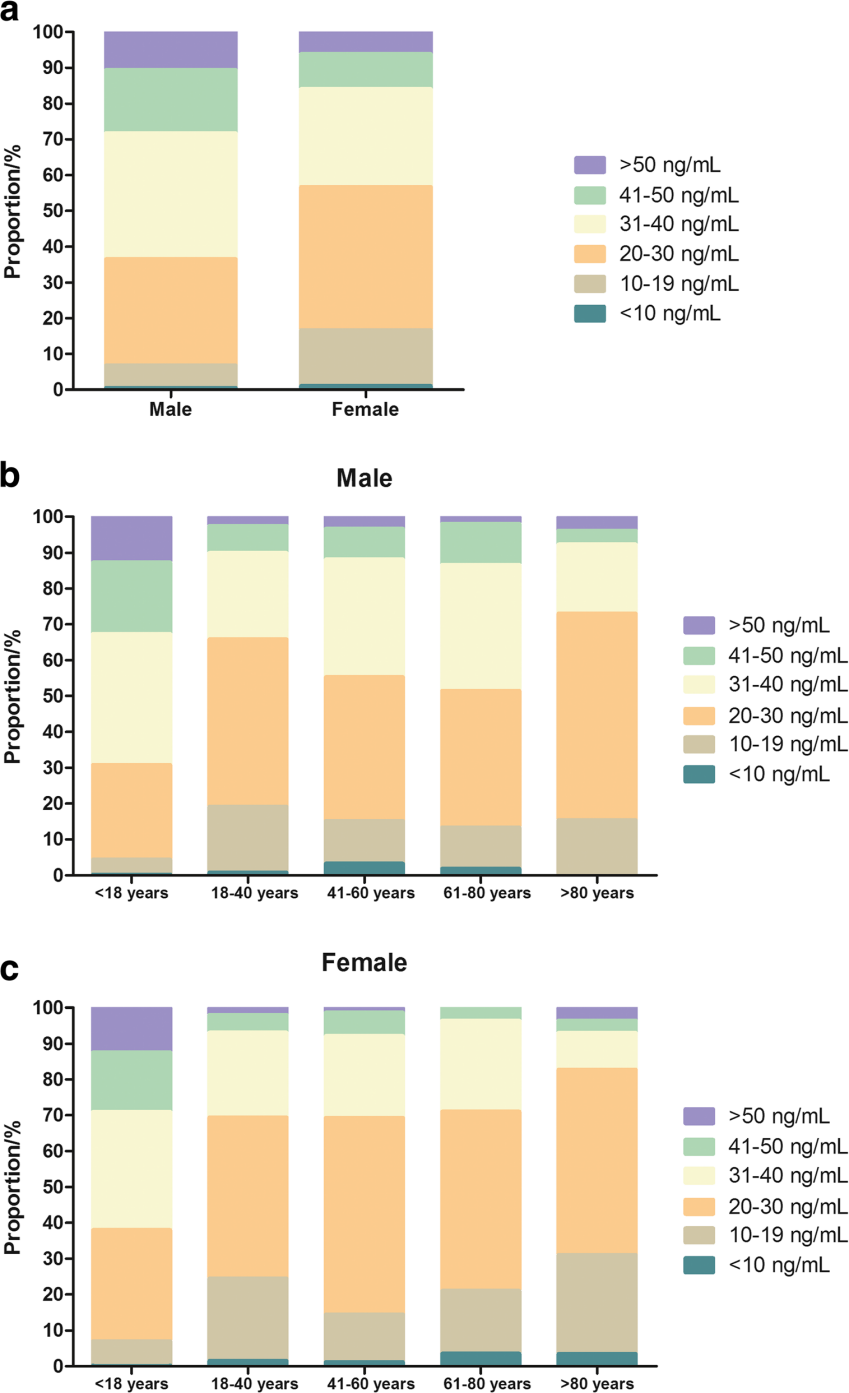
Distribution of serum 25(OH)D concentrations in all case (**a**), males (**b**) and females (**c**) of different age groups according to the categories recommended by the guidelines of the Endocrine Society

In males, the global 25(OH)D concentration was higher than in females of different ages (Table 5, median 34.26 vs. 29.40 ng/mL, *p* < 0.001). In particular, the 25(OH)D concentration in males within age groups (< 18 years: median 35.70 vs. 34.16 ng/mL, *p* < 0.01 and 61–80 years: median 30.50 vs. 27.20 ng/mL, p < 0.001) was significantly higher than that in females of the same age groups (Table 5). Figure 2 shows the frequency distribution of the total 25(OH)D results in the evaluated southern Chinese population. The classification of 25(OH)D results according to the functional categories suggested by the Endocrine Society and the proportion of 25(OH)D categories by age group is shown in Fig. 3.

The mean total 25(OH)D concentration was highest in males younger than 18 years and lowest in females older than 80 years (Table 5). The average 25(OH)D concentration showed no clear trend between age groups in males and females. An adequate total 25(OH)D concentration (> 30 ng/mL) was most frequently found in individuals aged < 18 years (61.97% of all women and 69.17% of all men, Fig. 3b-c). The prevalence of 25(OH)D deficiency (< 20 ng/mL) in females (16.65%) was higher than that in males (6.83%) (Fig. 3a). 25(OH)D deficiency was easily observed in men of 18–40 years of age (19.16%) and was common in women older than 80 years of age (31.04%) (Fig. 3b-c).

### Seasonal distribution of serum 25(OH)D concentrations in a South China population

**Fig. 4:**
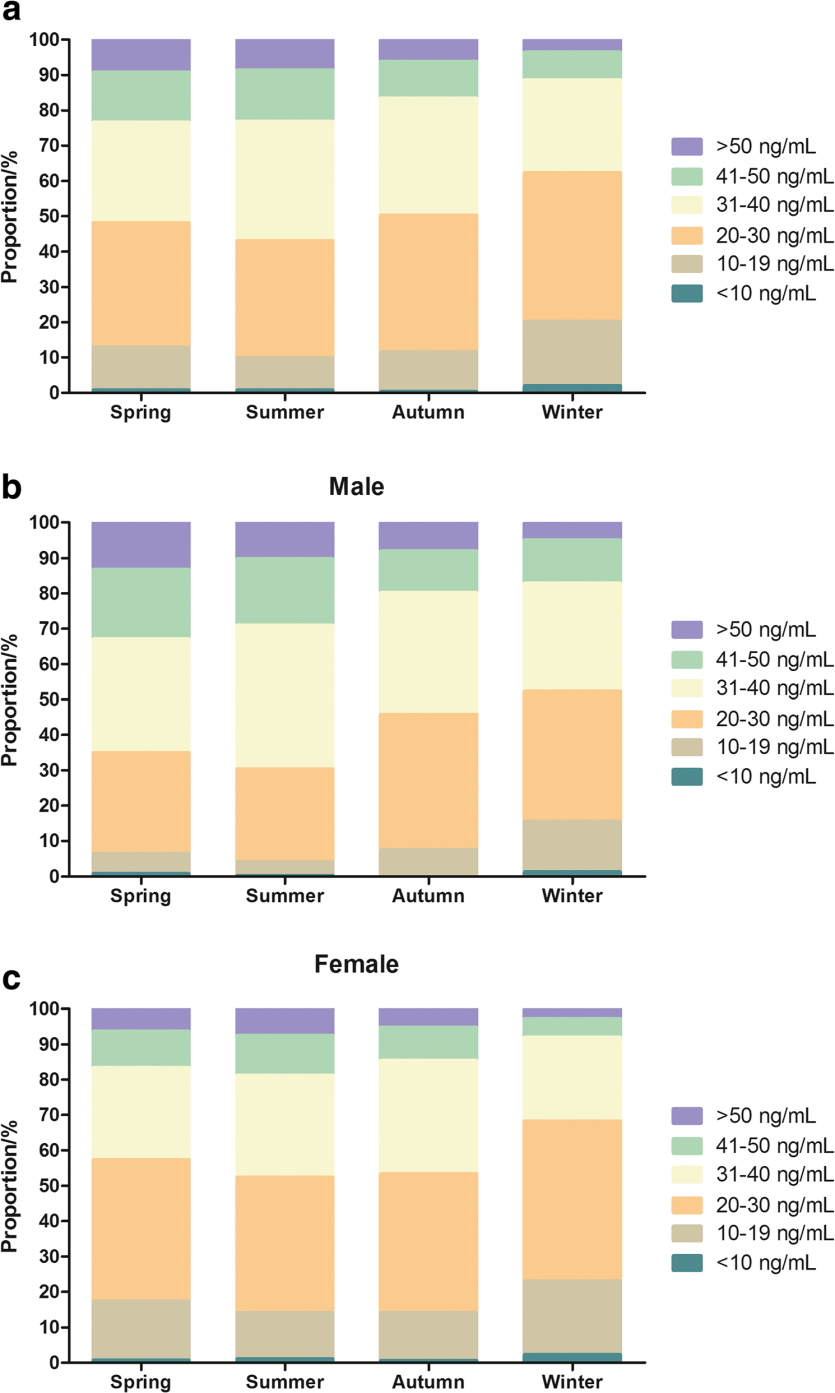
Seasonal distribution of serum 25(OH)D concentrations in all case (**a**), males (**b**) and females (**c**) according to the categories recommended by the guidelines of the Endocrine Society

**Fig. 5:**
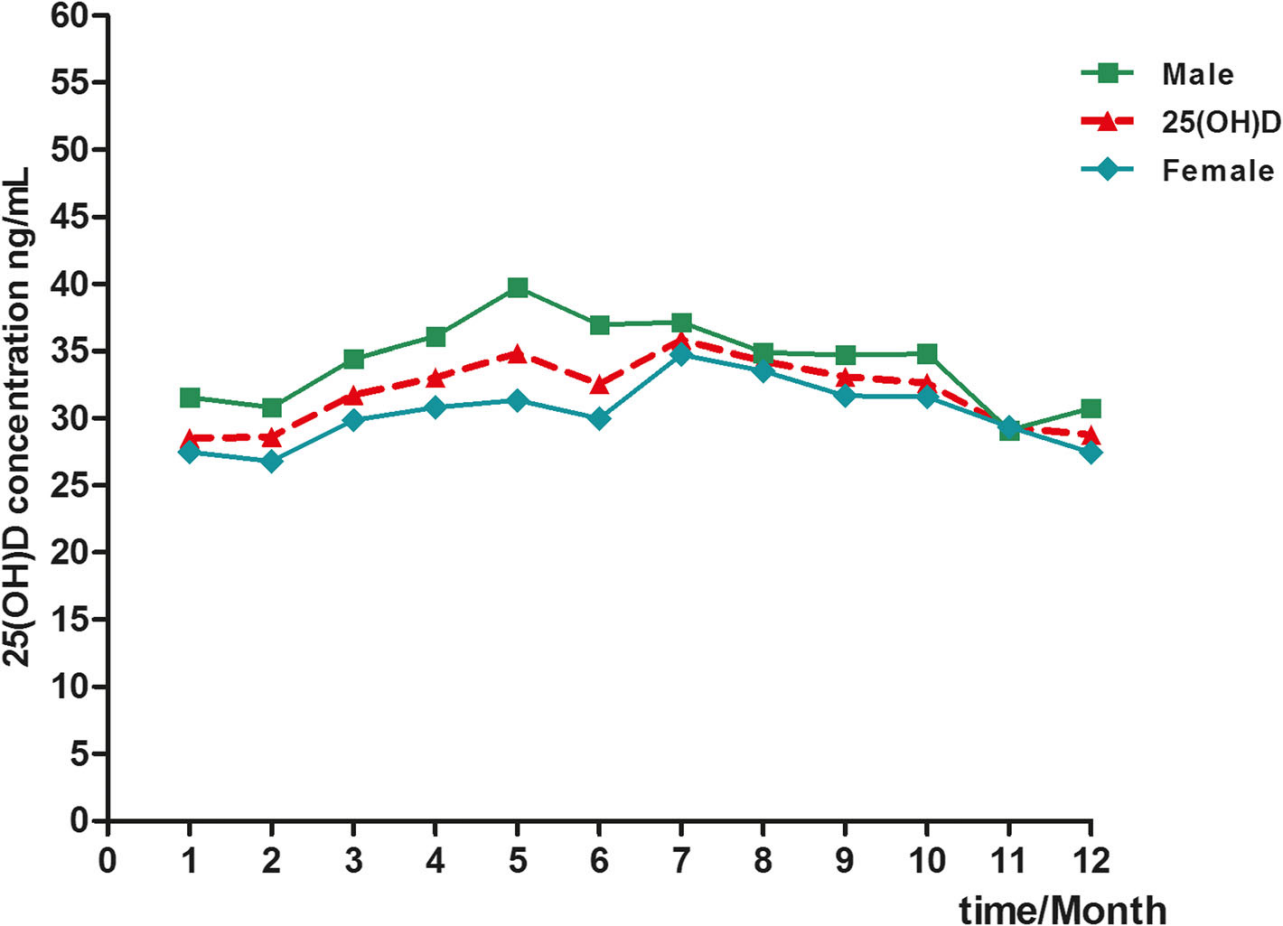
Distribution of serum 25(OH)D concentrations in cases, males and females during different seasons

The season-specific classification of 25(OH)D results according to the functional categories suggested by the Endocrine Society in males and females is presented in Fig. 4. The prevalence of 25(OH)D deficiency (< 20 ng/mL) per season was as follows: 12.83% in spring, 9.85% in summer, 11.75% in autumn, and 20.19% in winter (Fig. 4a). The prevalence of 25(OH)D deficiency (< 20 ng/mL) was most common in winter (22.98% of all women and 15.49% of all men, Fig. 4b-c). Plasma 25(OH)D concentrations were higher in subjects from whom blood samples were collected in summer and autumn than in those from whom blood samples were collected in winter and spring (Fig. 5).

**Fig. 6:**
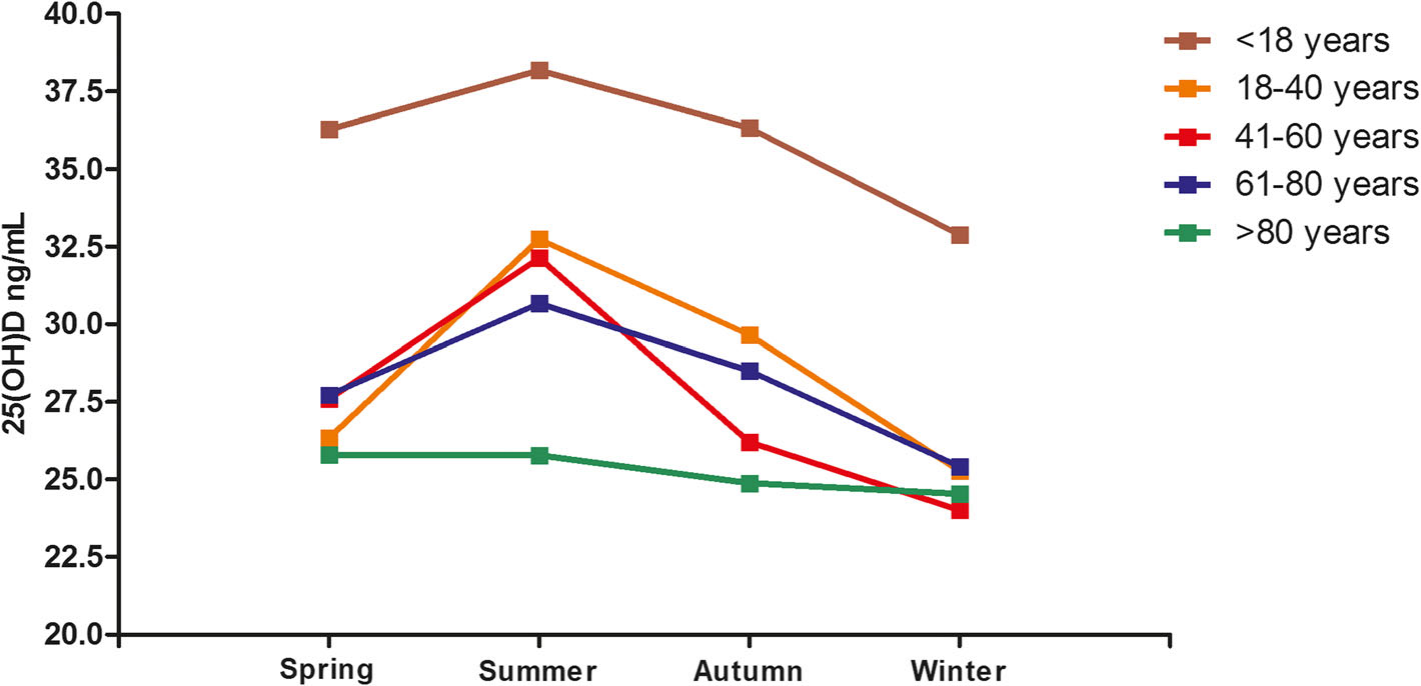
Seasonal variation of mean total serum 25(OH)D concentrations in different age groups

The degree of seasonal variation differed with age, as shown in Fig. 6. In individuals aged 41–60 years, the maximum seasonal change in serum 25(OH)D was 8.13 ng/mL, whereas in individuals > 80 years old, the trend was consistent (Fig. 6). The mean seasonal 25(OH)D concentration in the < 18 years of age group was significantly higher than that in the other groups (Fig. 6). In addition, the mean total 25(OH)D concentration was highest in summer in the different age groups (Fig. 6). The relationship between weather change and the 25(OH)D level in the evaluated South China population is shown in Table 6.

**Table 6:** The relationship between weather change and 25(OH)D levels in a population from South China

25(OH)D(ng/mL)	Spring			Summer			Autumn			Winter		
	March	April	May	June	July	August	September	October	November	December	January	February
Male	34.43 ± 11.10	36.08 ± 12.27	39.73 ± 12.28	36.94 ± 12.98	37.12 ± 9.81	34.89 ± 9.50	34.72 ± 11.12	34.80 ± 10.92	29.10 ± 14.16	30.74 ± 10.06	31.54 ± 11.79	30.81 ± 10.91
Female	29.85 ± 10.80	30.81 ± 11.98	31.35 ± 12.39	29.97 ± 11.91	34.74 ± 10.67	33.51 ± 10.91	31.67 ± 10.81	31.57 ± 11.13	29.36 ± 9.79	27.43 ± 10.04	27.46 ± 10.36	26.78 ± 9.25
Weather (days)												
Sunny	11	9.5	8	6	7	7	16	20	12	9	10.5	9
Overcast	9	8	10	7.5	8	9	5	4	8	12	10	11
Rain	11	12.5	13	16.5	16	15	9	7	10	10	10.5	8

### Prevalence of serum 25(OH)D_2_ in the evaluated population of South China

25(OH)D_2_ was detected in quantifiable concentrations by LC-MS/MS in 672 samples. This accounts for 14.07% of the entire study. 25(OH)D_2_ was found most frequently in the < 18 years group. The average 25(OH)D_2_ concentration in males and females was 9.41 and 7.93 ng/mL, respectively (*P* < 0.01, Table 7).

**Table 7:** Prevalence of serum 25 (OH) D_2_ in a population form South China

Sex	All		< 18 years	18-40 years	41-60 years	61-80 years	> 80 years
	M (*n* = 233)	F (*n* = 439)	*n* = 359	*n* = 230	*n* = 32	*n* = 38	*n* = 11
25(OH)D_2_(ng/mL)							
Median	6.50	6.06	7.20	5.67	4.33	3.15	7.47
Mean ± SD	9.41 ± 7.60	7.93 ± 6.42**	9.82 ± 7.67	6.84 ± 5.08	7.32 ± 6.52	5.39 ± 4.73	11.02 ± 9.29

*P < 0.05, **P < 0.01, *M* male, *F* female

### The correlation between concentrations of serum 25(OH)D and PTH

**Fig. 7:**
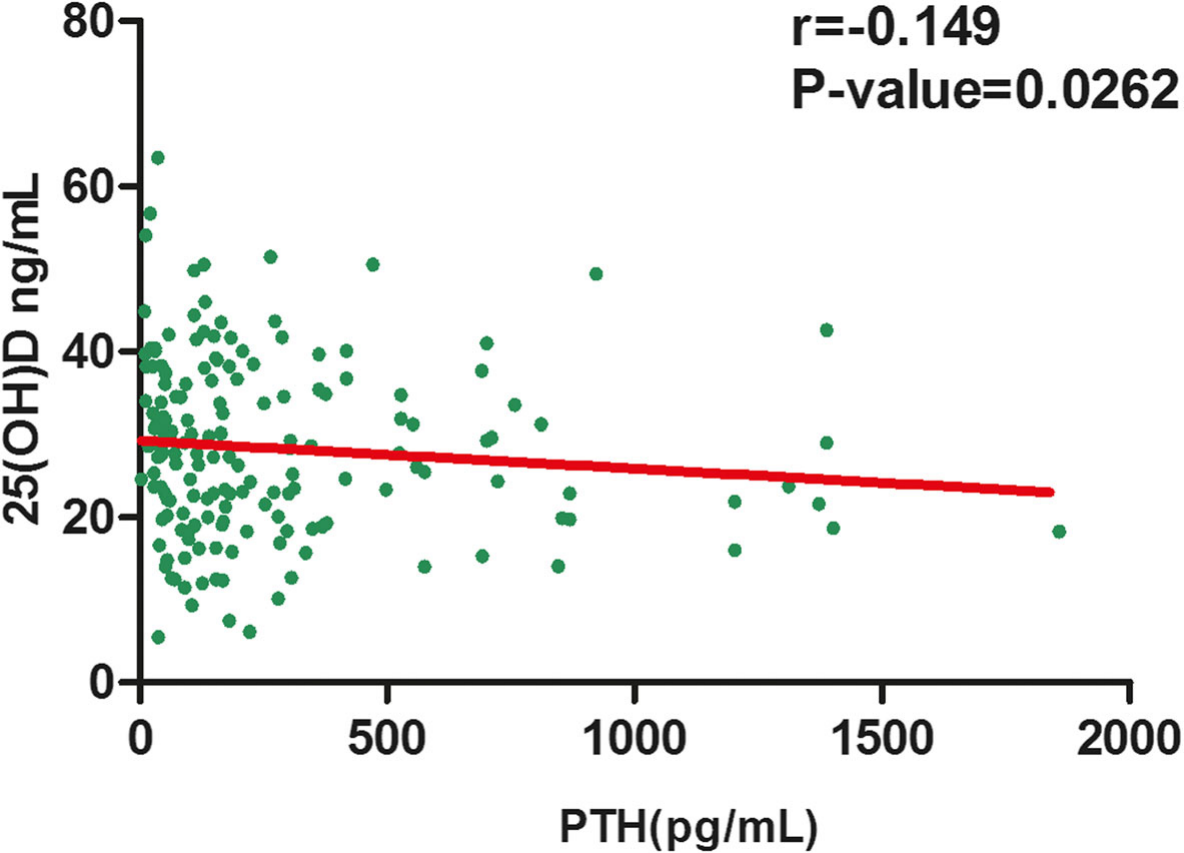
The correlation between concentrations of 25(OH)D and serum PTH in 162 samples

As shown in Table 8, we collected 162 samples with requested PTH detection from 4775 samples on the same occasion. The mean serum 25(OH)D concentration of the PTH-detected groups was lower than that of the entire cohort (Table 8. male: median 29.51 vs. 34.26 ng/mL, female: median 25.95 vs. 29.40 ng/mL). The PTH concentration in males was significantly higher than that in females (Table 8, *P* < 0.05). In addition, there was a negative correlation between the concentrations of 25(OH)D and serum PTH (Fig. 7, *r* = − 0.149, *P* < 0.05).

**Table 8:** PTH concentration in 170 serum samples from 4775 25(OH)D serum samples

Sex	All		18-40 years		41-60 years		61-86 years	
	M (*n* = 92)	F (*n* = 78)	M (*n* = 15)	F (*n* = 10)	M (*n* = 43)	F (*n* = 20)	M (*n* = 34)	F (*n* = 48)
PTH (pg/mL)								
Median	138.00	169.80*	98.42	343.10	136.30	288.85***	142.10	143.10
Mean ± SD	210.43 ± 243.62	339.65 ± 398.82	290.15 ± 401.12	703.45 ± 698.37	217.67 ± 237.09	461.46 ± 446.12	166.01 ± 137.40	213.109 ± 189.50
25(OH)D(ng/mL)								
Median	29.51	25.95	32.10	22.50	24.70	27.55	30.28	27.35
Mean ± SD	29.67 ± 11.32	26.86 ± 9.85	33.32 ± 12.77	24.90 ± 11.23	27.76 ± 11.36	26.87 ± 11.21	30.48 ± 10.41	27.26 ± 9.12

*P < 0.05, ***P < 0.001, *M* male, *F* female

## Discussion

Compared with other methods, the detection of serum 25(OH)D levels by LC-MS/MS showed better specificity and accuracy than those of other commonly utilized methods [16, 22]. In our study, we optimized a mass spectrometric method that meets the recommended RMP specifications for 25(OH)D metabolites to achieve optimal authenticity and precision. The LC-MS/MS method of our laboratory had high specificity and could detect 3-epi-25(OH)D_3_, 25(OH)D_3_ 3-epi-25(OH)D_2_ and 25(OH)D_2_ (Fig. 1). In addition, we evaluated the accuracy of the LC-MS/MS method by analysing SRM 972a, which showed that the relative error was within the allowable range (Tables 2 and 3). The 25(OH)D results determined by this LC-MS/MS method indicate that this method meets reference measurement procedure requirements. This study illustrated the 25(OH)D status, the prevalence of vitamin D deficiency and the correlation between concentrations of 25(OH)D and serum PTH in a South China population. To the best of our knowledge, this is the first study to focus on a large sample of individuals, including 4775 serum samples from South China. In our study, the mean 25(OH)D concentration of the study group was 32.57 ± 11.74 ng/mL (4.2–101.4 ng/mL). 25(OH)D was detected most frequently in children aged < 18 years (48.57%, Table 5). The high demand for 25(OH)D analysis in children may be due to increased awareness of the health problems associated with vitamin D deficiency and the high level of vitamins required for growth and development [23].

The mean 25(OH)D concentration (32.57 ng/mL) in this study was higher than that in individuals from northeast China (35.70 nmol/L equal to 14.28 ng/mL) [10]. These differences can be explained by the geographical location of the subjects, the amount of sunlight and the analytical methods used. The low concentration of 25(OH)D in individuals from northeastern China (38°N-56°N) is probably due to a lack of sunlight during long, cold and dark winters. In contrast, South China is located at a latitude of 23°N-34°N and has adequate annual sunshine [9]. In addition, vitamin supplementation may be one of the reasons for the differences between those studies [5]. Interestingly, the mean serum 25(OH)D level (32.57 ng/mL) for our group was higher than that in Hong Kong (28.30 ng/mL) [24]. This may be because rapid industrialization has exacerbated air pollution, resulting in a reduction in daily ultraviolet exposure (Hong Kong Observatory website addresses: http://www.hko.gov.hk or http://www.weather.gov.hk). In addition, the urbanization of Hong Kong has changed people’s ways of life, thereby reducing outdoor activities and sunlight exposure [25]. In other previous studies, serum 25(OH)D was determined by immunoassay techniques, and their analytical performances may be systematically different from that of the LC-MS/MS method used in our study.

The global 25(OH)D concentration in males was higher than that in females of different ages (Table 5). Feitong Wu et al. also found that the concentration of 25(OH)D in Chinese men was higher than that in women [26], and other studies also reported consistent results [27]. However, numerous studies showed that the 25(OH)D concentration in females was higher than that in males [8, 28] or that there was no gender difference in their studies [29, 30]. These differences may be due to different lifestyles, such as the duration of outdoor activities, different working environments and trace element supplementation [5]. It can be proposed that the differences between men and women are population-specific, and there may be no systematic differences in the 25(OH)D concentrations between genders.

According to the Endocrine Society, vitamin D deficiency is defined as a level of 25(OH)D lower than 20 ng/mL (50 nmol/L) [31, 32]. Vitamin D deficiency has been reported in several Asian countries in diverse study populations [5, 14, 33]. In our study, the prevalence of vitamin D deficiency (< 20 ng/mL) in females (16.65%) was higher than that in males (6.83%) (Fig. 3a). Jean Woo et al. also reported a similar rate of vitamin D deficiency (18%) among young women in Hong Kong but a higher rate of vitamin D deficiency (40%) in those from Beijing [9]. A great number of studies have estimated that more than 20% of U.S., Canadian and European women are vitamin D deficient [34, 35]. Some studies have shown that the general deficiency of vitamin D in women was related to their hormone levels, direct sunlight exposure and skin pigmentation [5, 36]. In addition, 25(OH)D deficiency was commonly observed in females over 80 years old (31.04%) (Fig. 3c), which is consistent with the results of Chapuy MC in French women. The main causes of this may be low calcium intake, and, to a lesser degree, age-related renal insufficiency. Moreover, vitamin D deficiency was more common in winter than in the other seasons (Fig. 4a). Various studies have also reported that the serum vitamin D levels in winter and spring in developing countries were lower than those in summer and autumn [5, 37]. The decrease in 25(OH)D levels in winter is mainly due to a lack of sunlight, with weaker ultraviolet radiation occurring during those months [7].

25(OH)D_2_ is one of the main interfering substances in the immunoassay of vitamin D [38]. Our results showed that the prevalence of quantifiable 25(OH)D_2_ was higher in our studied population than in a study from Southern Europe [8]. In addition, the average 25(OH)D_2_ concentration was higher in males than in females (Table 7, 9.41 vs. 7.93 ng/mL, *P* < 0.01). That may be because 25(OH)D_2_ supplements are often used [39]. Cashman et al. reported that age and vitamin D supplementation were positive predictors of 25(OH)D_2_ levels in serum [4]. Therefore, considering that many immunoassay methods cannot detect 25(OH)D_2_ and 25(OH)D_3_ equally, laboratories should take into account the prevalence of quantifiable concentrations of 25(OH)D_2_.

25(OH)D has an effect on bones, the intestines and kidneys, which stimulates calcium transmission from these organs to the blood. In addition, PTH can stimulate the production of 25(OH)D in the human body. A decrease in PTH depends on the negative feedback regulation of blood calcium and 25(OH)D [1]. This study indicated that there was a negative correlation between serum 25(OH)D and PTH levels (Fig. 7). Feitong Wu et al. also found that greater 25(OH)D was associated with reduced serum PTH levels in Chinese patients [26]. Other studies have shown a stronger correlation between 25(OH)D and PTH [40]. A mildly severe 25(OH)D deficiency can lead to an increase in serum PTH, which can cause bone resorption, osteoporosis and fractures. These results indicated that 25(OH)D plays an important role in inhibiting serum PTH, which can better protect bones.

## Conclusion

In conclusion, our study demonstrated that the average serum 25(OH)D concentration in individuals from southern China is higher than that in other Chinese cohorts by a high-accuracy LC-MS/MS method, which meets reference measurement procedure requirements. The global 25(OH)D concentration in males was higher than that in females of different ages, and the prevalence of vitamin D deficiency in females was higher than that in males. Seasonal change was an important factor of 25(OH)D concentration in young and middle-aged people but became less relevant in older subjects. 25(OH)D_2_ detection was of minor practical significance in our study. In addition, we also found that there was a negative correlation between the levels of serum 25(OH)D and PTH in individuals from southern China. However, further studies are required to confirm our findings.

## Supplementary information

**Additional file 1:** Experimental protocol of SRM 972a detection.

## Data Availability

The datasets used and/or analysed during the current study are available from the corresponding author on reasonable request.
