# Correction to: Determination of serum 25-hydroxyvitamin D status among population in southern China by a high accuracy LC-MS/MS method traced to reference measurement procedure

**DOI:** 10.1186/s12986-021-00558-z

**Published:** 2021-04-09

**Authors:** Zhiliang Cai, Qiaoxuan Zhang, Ziqiang Xia, Songbai Zheng, Lilan Zeng, Liqiao Han, Jun Yan, Peifeng Ke, Junhua Zhuang, Xinzhong Wu, Xianzhang Huang

**Affiliations:** 1 Department of Laboratory Medicine, The Second Affiliated Hospital of Guangzhou University of Chinese Medicine, Guangzhou, China; 2 Second Clinical Medical College, Guangzhou University of Chinese Medicine, Guangzhou, 510120 China; 3 Guangzhou Huayin Medical Laboratory Center, Guangzhou, China

## Correction to: Cai et al. Nutrition & Metabolism (2020) 17:8 https://doi.org/10.1186/s12986-020-0427-7

Following the publication of the original article [1], the authors identified errors in LC-MS/MS conditions section and Table 1. The changes have been highlighted in **bold typeface**.

**Table 1:** Conditions of triple quadrupole MS

Compound	Average mass	Precursor ion (m/z)	Preoduct ion (m/z)	Cone (V)	CE(V)
25(OH)VD_3_	400.6	383.3	257.2 (Q)	25	15
			365.1 (I)	24	15
**25(OH)VD**_**3**_**-**^**13**^**C**_**5**_	405.6	388.3	370.1	26	15
25(OH)VD_2_	412.7	395.3	119.4 (Q)	26	20
			377.3 (I)	26	10
**25(OH)VD**_**2**_**-d**_**3**_	415.7	398.3	380.3	26	15

*CE* collision energy, *Q* transition used for quantification, *I* transition used for identification

The sentence currently reads:

The selected reaction monitoring transitions were m/z 383.3 → 365.1 [25(OH)D3], m/z 383.3 → 257.2 [3-epi-25(OH)D3], m/z 386.3 → 368.1[25(OH)D3-d3)], m/z 395.3 → 119.3 [25(OH)D2], m/z 395.3 → 377.3[3-epi25(OH)D2] and m/z 398.3 → 380.3 [25(OH)D2-d2].

The sentence should read:

The selected reaction monitoring transitions were m/z 383.3 → 365.1 [25(OH)D_3_], m/z 383.3 → 257.2 [3-epi-25(OH)D_3_], **m/z 388.3 → 370.1[25(OH)VD**_**3**_**-**^**13**^**C**_**5**_**]**, m/z 395.3 → 119.3 [25(OH)D_2_], m/z 395.3 → 377.3[3-epi-25(OH)D_2_] and m/z 398.3 → 380.3 **[25(OH)VD**_**2**_**-d**_**3**_**]**.

The author group has been updated above and the original article [1] has been corrected.
