# Supplementary material for: Determination of serum 25-hydroxyvitamin D status among population in southern China by a high accuracy LC-MS/MS method traced to reference measurement procedure
Source: Nutr Metab (Lond). 2020 Jan 17;17:8. doi: 10.1186/s12986-020-0427-7 (PMC6969429; doi:10.1186/s12986-020-0427-7)
Supplement: Supplementary file 1 — Additional file 1: Experimental protocol of SRM 972a detection. [file 12986_2020_427_MOESM1_ESM.pdf]

National Institute of Standards & Technology

Certificate of Analysis

Standard Reference Material® 972a

Vitamin D Metabolites in Frozen Human Serum

This Standard Reference Material (SRM) is intended for use as an accuracy control in the critical evaluation of methods for determining the amount-of-substance concentration of vitamin D metabolites in human serum. This SRM can also be used as a quality assurance tool for assigning values to in-house control materials for these constituents. A unit of SRM 972a consists of four vials (Levels 1 through 4) of frozen serum with different concentration levels of 25-hydroxyvitamin D [25(OH)D] and 24R,25-dihydroxyvitamin D_3 [24R,25$(OH)_2D_3$]. Measurement of 25(OH)D in serum is generally considered a reliable indicator of vitamin D status. Measurement of 24R,25$(OH)_2D_3$ in serum is considered as a catabolism marker and an indicator of kidney disease. Each vial of SRM 972a contains approximately 1 mL of serum.

Each of the four levels of SRM 972a was prepared with specific target levels of 25(OH)D. While some measurement methods might be applicable to each of the four levels of SRM 972a, it is recognized that some methods may not be applicable to some levels. Individual users will need to assess which level or levels best suit their particular needs. Levels 1, 2, and 3 of SRM 972a were prepared from pools of human serum with endogenous concentrations of 25(OH)D. Level 4 was prepared from a pool of human serum that was fortified with 3-epi-25-hydroxyvitamin D_3.

Certified Values: The certified values for 25-hydroxyvitamin D_3 [25$(OH)D_3$], 25-hydroxyvitamin D_2 [25$(OH)D_2$], and 3-epi-25-hydroxyvitamin D_3 [3-epi-25$(OH)D_3$] are provided in Table 1. A NIST certified value is a value for which NIST has the highest confidence in its accuracy in that all known or suspected sources of bias have been investigated or taken into account [1]. The certified values for these analytes are based on the agreement of results from isotope dilution liquid chromatography mass spectrometry (ID-LC-MS) [2] and isotope dilution liquid chromatography tandem mass spectrometry (ID-LC-MS/MS) [3] procedures performed at NIST, and from ID-LC-MS/MS results provided by the Centers for Disease Control and Prevention (CDC), Atlanta, GA [4]. The NIST ID-LC-MS/MS method is recognized as a higher-order reference measurement procedure by the Joint Committee for Traceability in Laboratory Medicine (JCTLM) [5].

Reference Values: Reference values for 25$(OH)D_2$ and 3-epi-25$(OH)D_3$ are provided in Table 2. Reference values for 24R,25$(OH)_2D_3$ are provided in Table 3. Reference values are noncertified values that are the best estimate of the true values based on available data; however, the values do not meet the NIST criteria for certification and are provided with associated uncertainties that may reflect only measurement precision, may not include all sources of uncertainty, or may reflect a lack of sufficient statistical agreement among multiple analytical methods [1]. The reference values for 25$(OH)D_2$ and 3-epi-25$(OH)D_3$ are based on the agreement of results from ID-LC-MS and ID-LC-MS/MS procedures performed at NIST and from ID-LC-MS/MS results provided by the CDC. The reference values for 24R,25$(OH)_2D_3$ are based on the results from a candidate reference measurement procedure using ID-LC-MS/MS performed at NIST [6].

Expiration of Certification: The certification of **SRM 972a** is valid, within the measurement uncertainty specified, until **31 January 2023**, provided the SRM is handled and stored in accordance with the instructions given in this certificate (see "Instructions for Storage and Use"). The certification is nullified if the SRM is damaged, contaminated, or otherwise modified.

Maintenance of SRM Certificate: NIST will monitor this SRM over the period of its certification. If substantive technical changes occur that affect the certification before the expiration of this certificate, NIST will notify the purchaser. Registration (see attached sheet or register online) will facilitate notification.

Carlos A. Gonzalez, Chief
Chemical Sciences Division

Robert L. Watters, Jr., Director
Office of Reference Materials

Gaithersburg, MD 20899
Certificate Issue Date: 15 September 2015
Certificate Revision History on Last Page

Support for the development of SRM 972a was provided in part by the National Institutes of Health (NIH) Office of Dietary Supplements (ODS). Technical consultation was provided by C.T. Sempos, J.M. Betz and P.M. Coates of NIH-ODS.

Overall direction and coordination of the analytical measurements leading to the certification of this SRM were performed by K.W. Phinney of the NIST Biomolecular Measurement Division, and S. S.-C. Tai and L.C. Sander of the NIST Chemical Sciences Division.

Acquisition of the material was performed by K.W. Phinney and K.E. Sharpless of the NIST Office of Special Programs. Certification measurements were performed by M. Bedner and S.S.-C. Tai of the NIST Chemical Sciences Division and R.S.C. Chia, a guest scientist at NIST from the Health Sciences Authority of Singapore. Certification measurements were also performed by K. Maw, S. Encisco, M. Chaudhary-Webb, and R.L. Schleicher at the CDC. Additional measurements in support of the development of SRM 972a were performed by M.A. Nelson, B.E. Lang, M.M. Schantz, and L.T. Sniegoski of the NIST Chemical Sciences Division.

Statistical analysis was provided by J.H. Yen of the NIST Statistical Engineering Division.

Support aspects involved in the issuance of this SRM were coordinated through the NIST Office of Reference Materials.

NOTICE AND WARNINGS TO USERS

Warning: SRM 972a IS INTENDED FOR LABORATORY USE ONLY. THIS IS A HUMAN-SOURCE MATERIAL. HANDLE PRODUCT AS A BIOHAZARDOUS MATERIAL CAPABLE OF TRANSMITTING INFECTIOUS DISEASE. The supplier of the serum has reported that each donor unit of serum used in the preparation of this product has been tested by a FDA-approved method and found non-reactive/negative for hepatitis B surface antigen (HbsAg), human immunodeficiency (HIV) 1 and 2 antibodies, and hepatitis C virus (HCV). However, no known test method can offer complete assurance that hepatitis B virus, hepatitis C virus, HIV, or other infectious agents are absent from this material. Accordingly, this human blood-based product should be handled at the Biosafety Level 2 or higher as recommended for any POTENTIALLY INFECTIOUS HUMAN SERUM OR BLOOD SPECIMEN in the Centers for Disease Control/National Institutes of Health Manual [7].

INSTRUCTIONS FOR STORAGE AND USE

Storage: Until required for use, SRM 972a should be stored in the dark at a temperature between −20 °C and −80 °C.

Use: SRM 972a is provided as a set of four vials of frozen serum. The vial (or vials) to be used should be allowed to thaw at room temperature for at least 30 min under subdued light. The contents of the vial should then be gently mixed prior to removal of a test portion for analysis. Precautions should be taken to avoid exposure to strong UV light and direct sunlight.

SOURCE, PREPARATION, AND ANALYSIS[(1)]

Source and Preparation: SRM 972a was prepared by Solomon Park Research Laboratories (Kirkland, WA). Four serum pools were prepared. The naturally occurring concentrations of vitamin D metabolites in the human serum pools used to prepare Levels 1, 2, and 3 have not been modified. Level 4 is a human serum pool enriched with 3-epi-25(OH)D_3.

Analysis: Value assignment of the concentrations of 25(OH)D in SRM 972a was based on the combination of results provided from two analytical methods at NIST (ID-LC-MS and ID-LC-MS/MS) and from ID-LC-MS/MS at CDC. Value assignment of the concentrations of 24R,25$(OH)_2D_3$ are based on the results from ID-LC-MS/MS at NIST.

[(1)] Certain commercial equipment, instruments or materials are identified in this certificate to adequately specify the experimental procedure. Such identification does not imply recommendation or endorsement by the National Institute of Standards and Technology, nor does it imply that the materials or equipment identified are necessarily the best available for the purpose.

Measurement of 25(OH)D_3, 25(OH)D_2, and 3-epi-25(OH)D_3 by ID-LC-MS (NIST): Serum (450 mg) and an internal standard solution containing $^2H_6$-25(OH)D_3, $^2H_3$-25(OH)D_2, and $^2H_3$-3-epi-25(OH)D_3 were combined in glass tubes, proteins were precipitated, and the metabolites were extracted into hexane twice. The hexane phases were combined and evaporated to dryness at 40 °C under nitrogen. The residues were reconstituted with methanol and vortex-mixed. Extracts were analyzed by using LC-MS with (1) an Ascentis Express F5 pentafluorophenylpropyl column (Supelco, Bellefonte, PA) and (2) a Zorbax SB-CN cyanopropyl stationary phase (Agilent Technologies, Palo Alto, CA). Analyses on the pentafluorophenylpropyl column were performed under isocratic conditions with a mobile phase of 26 % water and 74 % methanol at a flow rate of 0.8 mL/min. All solvent compositions represent volume fractions in percent. The column temperature was maintained at 15 °C. A step gradient to 100 % methanol was incorporated into the method at the end of the run to elute retained matrix components. Analyses on the cyanopropyl column were performed under isocratic conditions with a mobile phase of 33 % water and 67 % methanol (Levels 1, 2, and 4 of SRM 972a) or 34 % water and 66 % methanol (Level 3) at a flow rate of 1.0 mL/min. A step gradient to 100 % methanol was incorporated into the method at the end of the run to elute retained matrix constituents. The column temperature was maintained at 45 °C.

Atmospheric pressure chemical ionization (APCI) with positive polarity was used for both chromatographic methods. The $[M - H_2O + H]^+$ ions were monitored and used for quantification of all species. The ions monitored included *m/z* 383 for 25(OH)D_3 and for 3-epi-25(OH)D_3; *m/z* 386 for $^2H_3$-3-epi-25(OH)D_3; *m/z* 389 for $^2H_6$-25(OH)D_3; *m/z* 395 for 25(OH)D_2; and *m/z* 398 for $^2H_3$-25(OH)D_2.

Measurement of 25(OH)D_3, 25(OH)D_2, and 3-epi-25(OH)D_3 by ID-LC-MS/MS (NIST): Serum (1.0 g to 2.0 g) was spiked with an appropriate internal standard solution [$^2H_6$-25(OH)D_3, $^2H_3$-25(OH)D_2, or $^2H_3$-3-epi-25(OH)D_3]. After equilibration at room temperature for 1 h, the pH of each sample was adjusted to pH 9.8 ± 0.2 with carbonate buffer. Analytes were extracted twice from the serum matrix with a mixture of hexane and ethyl acetate. The combined extracts were dried under nitrogen at 45 °C, and the residues were reconstituted with methanol for LC-MS/MS analysis. Extracts were analyzed using either an Ascentis Express F5 or a Zorbax SB-CN column under isocratic conditions with water:methanol mobile phases. APCI in the positive-ion mode and multiple reaction monitoring (MRM) mode were used. The following transitions were monitored: *m/z* 401 → *m/z* 383 for 25(OH)D_3 and 3-epi-25(OH)D_3; *m/z* 407 → *m/z* 389 for $^2H_6$-25(OH)D_3; *m/z* 404 → *m/z* 386 for $^2H_3$-3-epi-25(OH)D_3; *m/z* 413 → *m/z* 395 for 25(OH)D_2; and *m/z* 416 → *m/z* 398 for $^2H_3$-25(OH)D_2.

Measurement of 25(OH)D_3, 25(OH)D_2, and 3-epi-25(OH)D_3 by ID-LC-MS/MS (CDC): Samples of SRM 972a (100 µL) were spiked with the following isotopically-labeled internal standards: $^2H_6$-25(OH)D_3, $^2H_3$-25(OH)D_2, and $^2H_3$-epi-25(OH)D_3. Each serum sample was extracted using 1.5 mL hexane, and the supernatant was collected, dried under nitrogen at 25 °C, and reconstituted in 69 % methanol in water (volume fractions). Analytes were eluted from the extract (isocratic mobile phase same composition as the diluent) at a flow rate of 0.4 mL/min on a Thermo Hypersil GOLD pentafluorophenyl column at 28 °C and detected using APCI in positive-ion mode. Two transitions per vitamin D metabolite along with one transition per internal standard were monitored: 25(OH)D_3: *m/z* 383 → *m/z* 365 and *m/z* 383 → *m/z* 105; $^2H_6$-25(OH)D_3: *m/z* 389 → *m/z* 377; epi-25(OH)D_3: *m/z* 383 → *m/z* 365 and *m/z* 383 → *m/z* 105; $^2H_3$-epi-25(OH)D_3: *m/z* 386 → *m/z* 368; 25(OH)D_2: *m/z* 395 → *m/z* 377 and *m/z* 395 → *m/z* 209; $^2H_3$-25(OH)D_2: *m/z* 398 → *m/z* 380. Analytes were quantitated using six-point linear calibration curves traceable to SRM 2972 25-Hydroxyvitamin D2 and D3 Calibration Solutions, and internal standards were used to correct for recovery.

Measurement of 24R,25$(OH)_2D_3$ by a Candidate Reference Measurement Procedure using ID-LC-MS/MS (NIST): Serum (1.5 g to 2.0 g) was spiked with an appropriate internal standard solution ($^2H_6$-24R,25$(OH)_2D_3$). After equilibration at room temperature for 1 h, the pH of each sample was adjusted to pH 9.8 ± 0.2 with carbonate buffer. The 24R,25$(OH)_2D_3$ was extracted twice from the serum matrix with a mixture of hexane and ethyl acetate. The combined extracts were dried under nitrogen at 45 °C, and the residues were reconstituted with methanol for LC-MS/MS analysis. Extracts were analyzed using an Ascentis Express C_{18} column under isocratic conditions with a water:methanol mobile phase. APCI in the positive-ion mode and multiple reaction monitoring (MRM) mode were used. The following transitions were monitored: *m/z* 417 → *m/z* 381 for 24R,25$(OH)_2D_3$ and *m/z* 423 → *m/z* 387 for $^2H_6$-24R,25$(OH)_2D_3$.

Homogeneity Analysis: The homogeneity assessment was made at the time the certification analyses were performed. A stratified sampling plan was devised to test for homogeneity across the lot of vials. There was no apparent trend in the data when plotted against the sequence in which the vials were prepared, with the exception of 3-epi-25(OH)D_3 in Level 4. An additional component of uncertainty related to possible inhomogeneity has been included in the expanded uncertainty for this analyte in Level 4.

Certified Values for 25(OH)D: Values are weighted means of the results from analyses at NIST using ID-LC-MS and ID-LC-MS/MS and from CDC using ID-LC-MS/MS. The uncertainty provided with each certified value is an expanded uncertainty about the weighted mean to cover the measurand with approximately 95 % confidence; it incorporates Type B uncertainty components related to the analyses and expresses both the observed difference between the results from the methods and their respective uncertainties, consistent with the Guide to the Expression of Uncertainty in Measurement and with its Supplement 1 [8–11]. The expanded uncertainty is calculated as $U = ku_c$, where u_c is the combined uncertainty and k is a coverage factor corresponding to approximately 95 % confidence for each analyte [8]. For the certified values shown in Table 1, $k = 2$. The measurands are the amounts of substance listed in Table 1. Metrological traceability is to the SI derived units of mass fraction (expressed as ng/g), mass concentration (expressed as ng/mL), and amount-of-substance concentration (expressed as nmol/L).

Table 1. Certified Values for 25-hydroxyvitamin D in SRM 972a

	ng/g	ng/mL[(a)]	nmol/L[(b)]
**Level 1**			
25-hydroxyvitamin D_3	28.1 ± 1.1	28.8 ± 1.1	71.8 ± 2.7
3-epi-25-hydroxyvitamin D_3	1.77 ± 0.10	1.81 ± 0.10	4.5 ± 0.2
**Level 2**			
25-hydroxyvitamin D_2	0.80 ± 0.06	0.81 ± 0.06	2.0 ± 0.2
25-hydroxyvitamin D_3	17.7 ± 0.4	18.1 ± 0.4	45.1 ± 1.0
3-epi-25-hydroxyvitamin D_3	1.25 ± 0.09	1.28 ± 0.09	3.2 ± 0.2
**Level 3**			
25-hydroxyvitamin D_2	13.0 ± 0.3	13.3 ± 0.3	32.3 ± 0.8
25-hydroxyvitamin D_3	19.4 ± 0.4	19.8 ± 0.4	49.5 ± 1.1
**Level 4**			
25-hydroxyvitamin D_3	28.8 ± 0.9	29.4 ± 0.9	73.4 ± 2.3
3-epi-25-hydroxyvitamin D_3	25.4 ± 2.1	26.0 ± 2.2	64.8 ± 5.4

(a) Mass concentration levels were calculated from mass fractions using measured serum densities: Level 1, 1.02326 g/mL; Level 2, 1.02196 g/mL; Level 3, 1.02294 g/mL; and Level 4, 1.02295 g/mL.

(b) Molar concentration levels were calculated from mass concentration levels using the relative molecular masses. The relative molecular masses are 412.65 for 25(OH)D_2 and 400.64 for 25(OH)D_3 and 3-epi-25(OH)D_3. The equivalent conversion factors are 2.4234 for 25(OH)D_2 and 2.4960 for 25(OH)D_3 and 3-epi-25(OH)D_3.

Reference Values for 25(OH)D: Values are weighted means of the results from analyses at NIST using ID-LC-MS and ID-LC-MS/MS and from CDC using ID-LC-MS/MS. The measurands are the amounts of substance listed in Table 2 as determined by the indicated methods. Metrological traceability is to the SI derived units of mass fraction (expressed as ng/g), mass concentration (expressed as ng/mL), and amount-of-substance concentration (expressed as nmol/L). The uncertainty provided with each reference value is an expanded uncertainty about the weighted mean to cover the measurand with approximately 95 % confidence; it incorporates Type B uncertainty components related to the analyses and expresses both the observed difference between the results from the methods and their respective uncertainties, consistent with the ISO/JCGM Guide and with its Supplement 1 [8–11]. The expanded uncertainty is calculated as $U = ku_c$, where u_c is the combined uncertainty and k is a coverage factor corresponding to approximately 95 % confidence for each analyte [8]. For the reference values shown in Table 2, $k = 2$.

Table 2. Reference Values for 25-hydroxyvitamin D in SRM 972a

	ng/g	ng/mL[(a)]	nmol/L[(b)]
**Level 1**			
25-hydroxyvitamin D_2	0.52 ± 0.06	0.54 ± 0.06	1.3 ± 0.2
**Level 3**			
3-epi-25-hydroxyvitamin D_3	1.14 ± 0.14	1.17 ± 0.14	2.9 ± 0.4
**Level 4**			
25-hydroxyvitamin D_2	0.54 ± 0.10	0.55 ± 0.10	1.3 ± 0.2

(a) Mass concentration levels were calculated from mass fractions using measured serum densities: Level 1, 1.02326 g/mL; Level 2, 1.02196 g/mL; Level 3, 1.02294 g/mL; and Level 4, 1.02295 g/mL.

(b) Molar concentration levels were calculated from mass concentration levels using the relative molecular masses. The relative molecular masses are 412.65 for 25(OH)D_2 and 400.64 for 25(OH)D_3 and 3-epi-25(OH)D_3. The equivalent conversion factors are 2.4234 for 25(OH)D_2 and 2.4960 for 25(OH)D_3 and 3-epi-25(OH)D_3.

Reference Values for 24R,25(OH)$_2$D$_3$: Values are the method means of the results from analyses at NIST via a candidate reference measurement procedure using ID-LC-MS/MS. The measurands are the amounts of substance listed in Table 3 as determined by the indicated method. Metrological traceability is to the SI derived units of mass fraction (expressed as ng/g), mass concentration (expressed as ng/mL), and amount-of-substance concentration (expressed as nmol/L). The uncertainty provided with each reference value is an expanded uncertainty about the method mean to cover the measurand with approximately 95 % confidence; it incorporates Type B uncertainty components related to the analyses, consistent with the ISO/JCGM Guide [8]. The expanded uncertainty is calculated as $U = ku_c$, where u_c is the combined uncertainty and k is a coverage factor corresponding to approximately 95 % confidence for the analyte [8]. For the reference values shown in Table 3, $k = 2$.

Table 3. Reference Values for 24R,25-dihydroxyvitamin D_3 in SRM 972a

	ng/g	ng/mL[(a)]	nmol/L[(b)]
**Level 1**			
24R,25-dihydroxyvitamin D_3	2.60 ± 0.10	2.66 ± 0.10	6.38 ± 0.23
**Level 2**			
24R,25-dihydroxyvitamin D_3	1.38 ± 0.05	1.41 ± 0.05	3.39 ± 0.12
**Level 3**			
24R,25-dihydroxyvitamin D_3	1.58 ± 0.06	1.62 ± 0.06	3.88 ± 0.13
**Level 4**			
24R,25-dihydroxyvitamin D_3	2.58 ± 0.09	2.64 ± 0.09	6.32 ± 0.22

(a) Mass concentration levels were calculated from mass fractions using measured serum densities: Level 1, 1.02326 g/mL; Level 2, 1.02196 g/mL; Level 3, 1.02294 g/mL; and Level 4, 1.02295 g/mL.

(b) Molar concentration levels were calculated from mass concentration levels using the relative molecular mass. The relative molecular mass is 416.64 g/mol for 24R,25(OH)$_2$D$_3$. The equivalent conversion factor is 2.4002.

Certified and Reference Values for Total 25(OH)D: Vitamin D levels in serum are typically reported as the total of $25(OH)D_3$ and $25(OH)D_2$. The values for total 25(OH)D, as the sum of the individual values for $25(OH)D_3$ and $25(OH)D_2$, are shown in Table 4 for certified values and Table 5 for reference values. The uncertainty provided with each value is an expanded uncertainty about the total 25(OH)D to cover the measurands with approximately 95 % confidence; it incorporates Type B uncertainty components related to the analyses and their respective uncertainties of the two analytes, consistent with the ISO/JCGM Guide and its Supplement 1 [8–11]. The expanded uncertainty is calculated as $U = ku_c$, where u_c is the combined uncertainty and k is a coverage factor corresponding to approximately 95 % confidence for the analytes [8]. For the values shown in Table 4 and Table 5, $k = 2$.

Table 4. Certified Values for Total 25(OH)D in SRM 972a[(a)]

	ng/g			ng/mL[(b)]		
**Level 2**						
total 25(OH)D	18.5	±	0.4	18.9	±	0.4
**Level 3**						
total 25(OH)D	32.4	±	0.5	33.2	±	0.5

[(a)] Certified values for total 25(OH)D are based on the combination of certified values for both $25(OH)D_2$ and $25(OH)D_3$. The measurands are the amounts of substance listed in Table 4. Metrological traceability is to the SI derived units of mass fraction (expressed as ng/g) and mass concentration (expressed as ng/mL).
[(b)] Mass concentration levels were calculated from mass fractions using measured serum densities: Level 1, 1.02326 g/mL; Level 2, 1.02196 g/mL; Level 3, 1.02294 g/mL; and Level 4, 1.02295 g/mL.

Table 5. Reference Values for Total 25(OH)D in SRM 972a[(a)]

	ng/g			ng/mL[(b)]		
**Level 1**						
total 25(OH)D	28.7	±	1.1	29.3	±	1.1
**Level 4**						
total 25(OH)D	29.3	±	0.9	30.0	±	0.9

[(a)] Reference values for total 25(OH)D are based on the combination of a reference value for $25(OH)D_2$ and a certified value for $25(OH)D_3$. The measurands are the amounts of substance listed in Table 5 as determined by the indicated method. Metrological traceability is to the SI derived units of mass fraction (expressed as ng/g) and mass concentration (expressed as ng/mL).
[(b)] Mass concentration levels were calculated from mass fractions using measured serum densities: Level 1, 1.02326 g/mL; Level 2, 1.02196 g/mL; Level 3, 1.02294 g/mL; and Level 4, 1.02295 g/mL.

REFERENCES

[1] May, W.; Parris, R.; Beck II, C.; Fassett, J.; Greenberg, R.; Guenther, F.; Kramer, G.; Wise, S.; Gills, T.; Colbert, J.; Gettings, R.; MacDonald, B.; *Definition of Terms and Modes Used at NIST for Value-Assignment of Reference Materials for Chemical Measurements*; NIST Special Publication 260-136 (2000); available at http://www.nist.gov/srm/publications.cfm (accessed Sep 2015).

[2] Bedner, M.; Phinney, K.W.; *Development and Comparison of Three Liquid Chromatography–Atmospheric Pressure Chemical Ionization/Mass Spectrometry Methods for Determining Vitamin D Metabolites in Human Serum*; J. Chromatogr. A, Vol. 1240, pp. 132–139 (2012).

[3] Tai, S.S.-C.; Bedner, M.; Phinney, K.W.; *Development of a Candidate Reference Measurement Procedure for the Determination of 25-Hydroxyvitamin D_3 and 25-Hydroxyvitamin D_2 in Human Serum Using Isotope-Dilution Liquid Chromatography–Tandem Mass Spectrometry*; Anal. Chem., Vol. 82, pp. 1942–1948 (2010).

[4] Schleicher, R.L.; Encisco, S.; Chaudhary-Webb, M.; Paliakov, E.; McCoy, L.F.; Pfeiffer, C.M.; *Isotope-Dilution Ultra Performance Liquid Chromatography–Tandem Mass Spectrometry Method for Simultaneous Measurement of 25-Hydroxyvitamin D_2, 25-Hydroxyvitamin D_3 and 3-epi-25-Hydroxyvitamin D_3 in Human Serum*; Clin. Chim. Acta, Vol. 412, pp. 1594–1599 (2011).

[5] Joint Committee for Traceability in Laboratory Medicine (JCTLM); available at http://www.bipm.org/en/committees/jc/jctlm/ (accessed Sep 2015).

[6] Tai, S.S.-C.; Nelson, M.A.; *Candidate Reference Measurement Procedure for the Determination of 24R,25-Dihydroxyvitamin D3 in Human Serum using Isotope-Dilution Liquid Chromatography-Tandem Mass Spectrometry*; Anal. Chem., Vol. 87 (15), pp 7964–7970 (2015).

[7] CDC/NIH; *Biosafety in Microbiological and Biomedical Laboratories, 5th ed.*; Richardson, J.; Barkley, W.E.; Richmond, J.; McKinney, R.W., Eds.; U.S. Department of Health and Human Services, Public Health Service, Centers for Disease Control and Prevention and National Institutes of Health; US Government Printing Office: Washington, D.C. (2009); available at http://www.cdc.gov/OD/OHS/biosfty/bmbl5/BMBL_5th_Edition.pdf (accessed Sep 2015).

[8] JCGM 100:2008; *Evaluation of Measurement Data — Guide to the Expression of Uncertainty in Measurement* (GUM 1995 with Minor Corrections); Joint Committee for Guides in Metrology (2008); available at http://www.bipm.org/utils/common/documents/jcgm/JCGM_100_2008_E.pdf (accessed Sep 2015); see also Taylor, B.N.; Kuyatt, C.E.; *Guidelines for Evaluating and Expressing the Uncertainty of NIST Measurement Results*; NIST Technical Note 1297; U.S. Government Printing Office: Washington, DC (1994); available at http://www.nist.gov/pml/pubs/tn1297/index.cfm (accessed Sep 2015).

[9] JCGM 101:2008; *Evaluation of Measurement Data – Supplement 1 to the Guide to the Expression of Uncertainty in Measurement – Propagation of Distributions Using a Monte Carlo Method*; JCGM (2008); available at http://www.bipm.org/utils/common/documents/jcgm/JCGM_101_2008_E.pdf (accessed Sep 2015).

[10] DerSimonian, R.; Laird, N.; *Meta-Analysis in Clinical Trials*; Controlled Clin. Trials, Vol. 7, pp. 177–188 (1986).

[11] Rukhin, A. L.; *Weighted Means Statistics in Interlaboratory Studies*; Metrologia, Vol. 46, pp. 323–331 (2009).

Certificate Revision History: 15 September 2015 (Addition of reference values for 24R,25$(OH)_2D_3$; addition of certified and reference values for total 25(OH)D; update of certified and reference values for 25$(OH)D_3$ and 3-epi-25$(OH)D_3$; editorial changes); 20 February 2013 (Original certificate issue date).

Users of this SRM should ensure that the Certificate of Analysis in their possession is current. This can be accomplished by contacting the SRM Program: telephone (301) 975-2200; fax (301) 948-3730; e-mail srminfo@nist.gov; or via the Internet at http://www.nist.gov/srm.
